# Draft Genome Sequence of *Bacillus amyloliquefaciens* XK-4-1, a Plant Growth-Promoting Endophyte with Antifungal Activity

**DOI:** 10.1128/genomeA.01306-15

**Published:** 2015-11-12

**Authors:** Zhengxiang Sun, Tom Hsiang, Yi Zhou, Jinglong Zhou

**Affiliations:** a College of Agriculture, Yangtze University, Jingzhou, Hubei, China; b Engineering Research Center of Ecology and Agricultural Use of Wetland, Ministry of Education, Jingzhou, Hubei, China; c Environmental Sciences, University of Guelph, Guelph, Ontario, Canada

## Abstract

Here, we report the draft genome sequence of a bacterial plant-growth-promoting endophyte, *Bacillus amyloliquefaciens* XK-4-1, which consists of one circular chromosome of 3,941,805 bp with 3,702 coding sequences (CDSs). The data presented highlight multiple sets of functional genes associated with its plant-beneficial characteristics.

## GENOME ANNOUNCEMENT

*Bacillus amyloliquefaciens* is considered to be a member of plant-beneficial bacteria, is known to promote plant growth and suppress plant pathogens, and has been applied widely in agriculture (1). This organism has been shown to produce a number of compounds with antifungal activity (2). *B. amyloliquefaciens* XK-4-1 was isolated from cotton (*Gossypium* spp.) during the flower bud formation stage and was found to have high levels of epiphytic populations during this stage. Our previous work showed that *B. amyloliquefaciens* XK-4-1 provides efficacious control of *Verticillium* wilt of cotton, in both pot experiment and field tests. Here, we report the draft genome sequence of XK-4-1. The genome sequence will facilitate further studies on the secondary metabolite biosynthetic pathways and broaden the current understanding of the biological control mechanism of *B. amyloliquefaciens* XK-4-1.

Genomic DNA of *B. amyloliquefaciens* XK-4-1 was extracted (3) and sequenced at Génome Québec, Montreal, Quebec, Canada, using Illumina HiSeq technology. The data were assembled using the freely available software ABySS (4), SOAP*denovo* (5), and Velvet (6). Gaps between contigs were closed by using GapCloser version 1.12. Annotation was performed using SEED and the Rapid Annotations using Subsystems Technology (RAST) (7, 8).

Analysis of the *B. amyloliquefaciens* XK-4-1 genome revealed that it consists of a circular chromosome of 3,941,805 bp (about 887× coverage depth; *N*_50_, 313,045 bp) in size, with 3,702 coding sequences (CDSs), 82 tRNA genes, 1 noncoding RNA (ncRNA), and 9 rRNAs, and an average G+C content of 46.0%. Some of these predicted genes and functions are known to be associated with growth promotion and biocontrol and are considered to play important roles in the biocontrol of some fungal pathogens. These include genes encoding chemotaxis-related proteins, sporulation transcription factors related to biofilm formation, antimicrobial biosynthesis, bacteriocins, siderophore-synthesis, and production of endoglucanases, cellulases, and glucanases.

### Nucleotide sequence accession numbers.

All raw and assembled data of *B. amyloliquefaciens* XK-4-1 for the project have been deposited at DDBJ/EMBL/GenBank under the accession no. LJDI00000000. The version described in this paper is version LJDI01000000.
